# Supplementary material for: Immunoinformatics Design and Identification of B-Cell Epitopes from Vespa affinis PLA1 Allergen
Source: Toxins (Basel). 2025 Jul 28;17(8):373. doi: 10.3390/toxins17080373 (PMC12390063; doi:10.3390/toxins17080373)
Supplement: Supplementary file 1 [file toxins-17-00373-s001.zip › toxins-3731325-supplementary.pdf]

toxins

MDPI

Supplementary Materials

Immunoinformatics Design and Identification of B-Cell Epitopes from *Vespa affinis* PLA1 Allergen

Sophida Sukprasert, Siriporn Nonkhwao, Thitijchaya Thanwiset, Walter Keller and Sakda Daduang

A

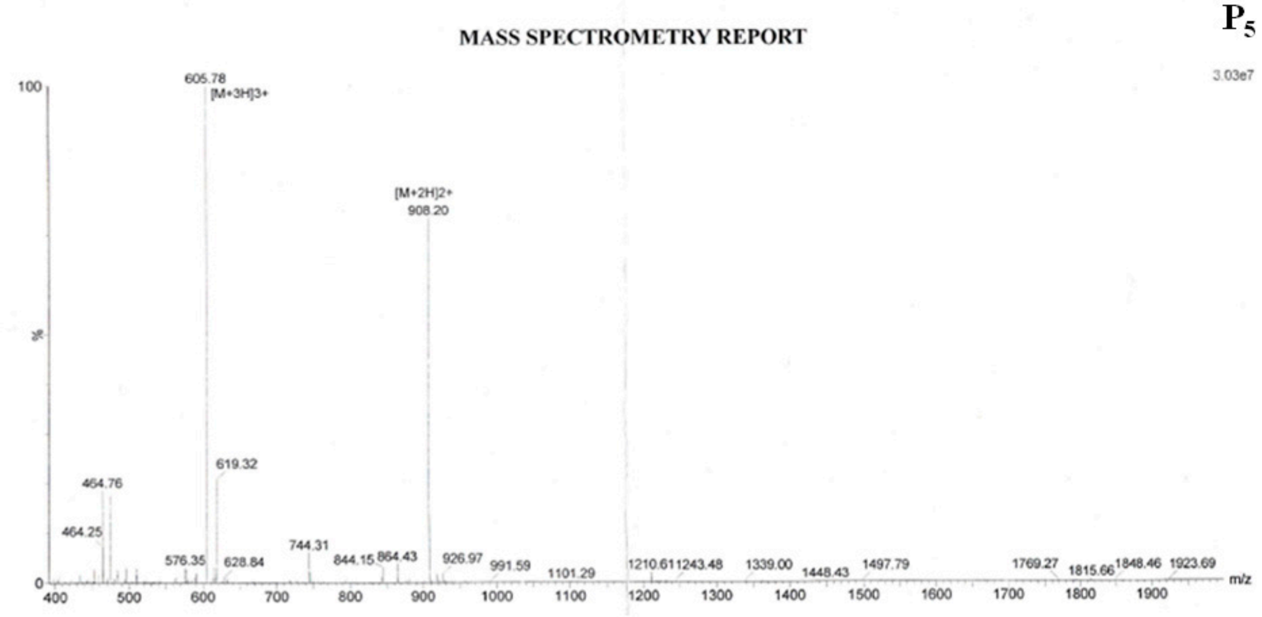

B

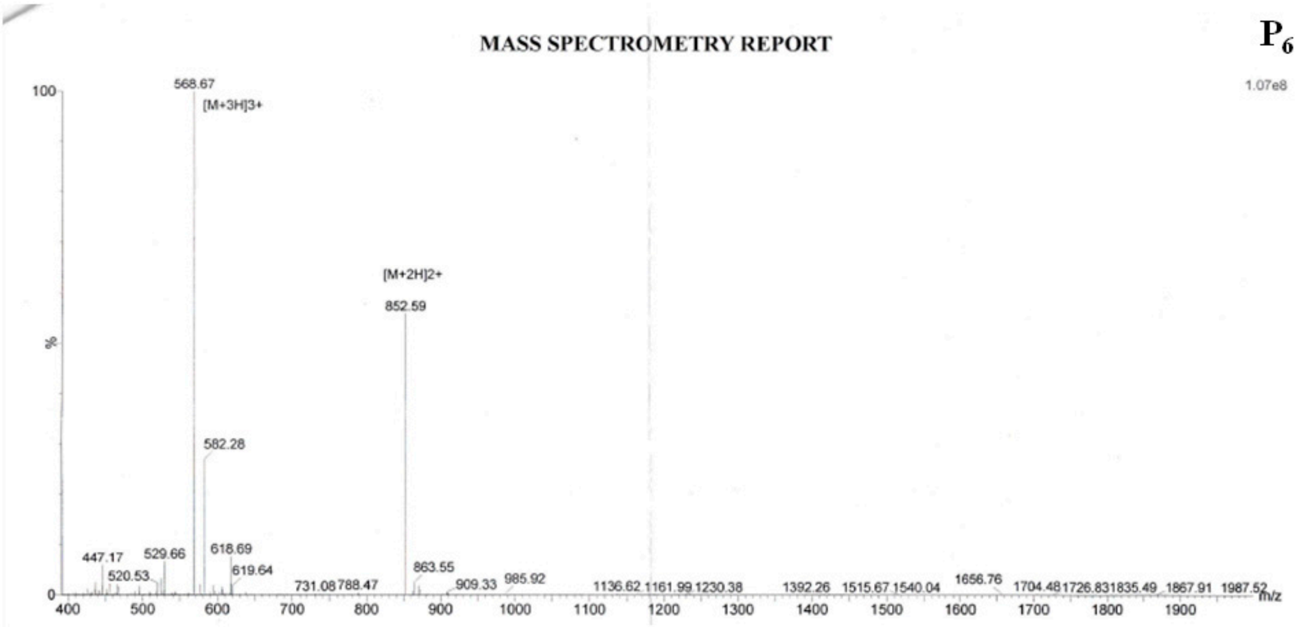

Figure S1. (**A**,**B**) Molecular mass determination of epitope peptides P5 and P6 by ESI-MS.

 https://doi.org/10.3390/toxins17080373

Table S1. Mean gray values of dot blot inhibitory activity for synthetic peptides EP5 and EP6, incubated with pAb-nVes a 1 and pAb-rVes a 1 antisera, were measured using ImageJ

Polyclonal antibody	Peptide/Protein Epitopes	Mean gray value[a]
pAb-nVes a 1 antisera	nVes a 1 (negative)	203.85
	nVes a 1 + EP5	213.45
	nVes a 1 + EP6	215.16
	nVes a 1 + nVes a 1 (positive)	219.79
	rVes a 1 (negative)	166.24
	rVes a 1 + EP5	171.54
	rVes a 1 + EP6	177.98
	rVes a 1 + rVes a 1 (positive)	193.69
pAb-rVes a 1 antisera	nVes a 1 (negative)	248.93
	nVes a 1 + EP5	245.07
	nVes a 1 + EP6	242.85
	nVes a 1 + nVes a 1 (positive)	239.13
	rVes a 1 (negative)	99.29
	rVes a 1 + EP5	143.89
	rVes a 1 + EP6	159.51
	rVes a 1 + rVes a 1 (positive)	187.75

[a]Low mean gray value is darker area (higher staining or band intensity), while a high mean gray value means a lighter area (lower staining or band intensity).
